# The PERFORM Study: Artificial Intelligence Versus Human Residents in Cross-Sectional Obstetrics-Gynecology Scenarios Across Languages and Time Constraints

**DOI:** 10.1016/j.mcpdig.2025.100206

**Published:** 2025-03-08

**Authors:** Canio Martinelli, Antonio Giordano, Vincenzo Carnevale, Sharon Raffaella Burk, Lavinia Porto, Giuseppe Vizzielli, Alfredo Ercoli

**Affiliations:** a Sbarro Institute for Cancer Research and Molecular Medicine and Center of Biotechnology, College of Science and Technology, Temple University, Philadelphia, PA; b Department of Human Pathology of Adult and Childhood “Gaetano Barresi,” Unit of Obstetrics and Gynecology, University of Messina, Messina, Italy; c Department of Medical Biotechnology, University of Siena, Siena, Italy; d Institute for Computational Molecular Science, Temple University, Philadelphia, PA; e Department of Medicine (DAME), Università degli Studi di Udine, Udine, Italy

## Abstract

**Objective:**

To systematically evaluate the performance of artificial intelligence (AI) large language models (LLMs) compared with obstetrics-gynecology residents in clinical decision-making, examining diagnostic accuracy and error patterns across linguistic domains, time constraints, and experience levels.

**Patients and Methods:**

In this cross-sectional study, we evaluated 8 AI LLMs and 24 obstetrics-gynecology residents (Years 1-5) using 60 standardized clinical scenarios. Most AI LLMs and all residents were assessed in May 2024, whereas chat GPT-01-preview, chat-GPT4o, and Claude Sonnet 3.5 were evaluated in November 2024. The assessment framework incorporated English and Italian scenarios under both timed and untimed conditions, along with systematic error pattern analysis. The primary outcome was diagnostic accuracy; secondary end points included AI system stratification, resident progression, language impact, time pressure effects, and integration potential.

**Results:**

The AI LLMs reported superior overall accuracy (73.75%; 95% confidence interval [CI], 69.64%-77.49%) compared with residents (65.35%; 95% CI, 62.85%-67.76%; *P*<.001). High-performing AI systems (ChatGPT-01-preview, GPT4o, and Claude Sonnet 3.5) achieved consistently high cross-linguistic accuracy (88.33%) with minimal language impact (6.67%±0.00%). Resident performance declined significantly under time constraints (from 73.2% to 56.5% adjusted accuracy; Cohen’s d=1.009; *P*<.001), whereas AI systems reported lesser deterioration. Error pattern analysis indicated a moderate correlation between AI and human reasoning (r=0.666; *P*<.001). Residents exhibited systematic progression from year 1 (44.7%) to year 5 (87.1%). Integration analysis found variable benefits across training levels, with maximum enhancement in early-career residents (+29.7%; *P*<.001).

**Conclusion:**

High-performing AI LLMs reported strong diagnostic accuracy and resilience under linguistic and temporal pressures. These findings suggest that AI-enhanced decision-making may offer particular benefits in obstetrics and gynecology training programs, especially for junior residents, by improving diagnostic consistency and potentially reducing cognitive load in time-sensitive clinical settings.

Artificial intelligence (AI) is reshaping health care, with large language models (LLMs) achieving accuracies exceeding 80% on standardized medical licensing examinations.^1^^,^^2^ Despite such impressive performance, questions remain regarding how these systems approach complex clinical decision-making particularly in obstetrics and gynecology (OB-GYN). Although LLMs can generate coherent responses through deep learning architectures, their internal reasoning processes remain largely opaque^3^ and they can produce misleading hallucinations that are difficult to detect without expert oversight.^4^^,^^5^

Recent research has highlighted both the promise and limitations of AI in health care settings, whereas AI tools have shown impressive performance in structured tasks and knowledge assessment.^6^, ^7^, ^8^ But their integration into real-world clinical practice requires careful evaluation, and it has to be on the basis of the reliability across different contexts and on the ability to complement rather than replace human clinical expertise.^9^^,^^10^ Existing literature highlights AI’s promise in various OB-GYN applications^11^^,^^12^ yet, no studies have systematically compared LLM performance with clinicians under conditions such as language variation, time pressure, and different levels of clinical expertise. Moreover, there is a lack of data on error patterns and the consequences of integrating AI into routine practice. Addressing these gaps is essential to ensure that AI augments, rather than undermines, clinical decision-making.^13^^,^^14^ This study examines 3 key dimensions of clinical decision-making: diagnostic accuracy across experience levels, cross-linguistic performance in standardized scenarios, and decision quality under temporal constraints. Through comprehensive analysis of error patterns, cognitive processes, and integration potential, we provide empirical insights into the complementary roles of human expertise and AI in OB-GYN care. This research contributes vital empirical evidence to inform the implementation of AI-augmented clinical practice in women’s health care, with particular emphasis on educational integration and decision support applications.^15^

## Methods

### Study Design

We conducted a cross-sectional comparative analysis evaluating the performance of AI language models and human medical residents in responding to standardized clinical scenarios following STROBE guideline. Detailed development processes for these scenarios are provided in Supplemental Document 1 (available online at https://www.mcpdigitalhealth.org/): clinical case scenario and STROBE guideline.

### Population

The study population comprised 24 OB-GYN residents from the OB-GYN School at University of Messina, distributed across training levels: first year (n=5, 20.8%), second year (n=7, 29.2%), and equal representation of third-year, fourth-year, and fifth-year residents (n=4, 16.7% each). To ensure standardized linguistic competency for bilingual assessment, all participating residents were native Italian speakers, and they had successfully completed the Medical English Proficiency Examination at the University of Messina. This institution is recognized in the World Directory of Medical Schools, with accreditation from both the Canadian medical regulatory authorities and the American Educational Commission for Foreign Medical Graduates.

For technological comparison, we evaluated 8 widely accessible LLMs: Meta AI, Google Gemini, OpenAI’s models (GPT-3.5, GPT-4, GPT-4 Mini, and ChatGPT-01 preview), and Anthropic’s variants (Claude 3.5 Sonnet and Claude 3.0 Haiku). Model selection prioritized systems with global accessibility and popularity.

### Procedure

The assessment framework utilized 60 multiple-choice clinical scenarios designed to evaluate 2 key parameters: the impact of time pressure and the influence of language on clinical reasoning performance. To ensure systematic evaluation, we implemented a balanced distribution protocol across both temporal and linguistic dimensions. Forty scenarios were administered without time constraints, whereas 20 scenarios incorporated specific time limitations. Concurrently, we maintained equal representation of English (30 scenarios) and Italian (30 scenarios) across both temporal conditions.

### Test Administration

The assessment protocol employed distinct methodologies for human and AI evaluation. The resident assessment was conducted in a controlled environment with proctored examination conditions. The AI system evaluation was performed by an OB-GYN specialist using consumer-grade hardware in Philadelphia, Philadelphia, across 2 phases (May 2024 and November 2024), utilizing standardized prompting protocols to ensure response consistency.

Detailed testing procedures, environmental conditions, and prompting protocols are provided in Supplemental Document 2 (available online at https://www.mcpdigitalhealth.org/): test administration and response collection protocol.

### Data Collection

All human residents’ answers were automatically collected through the platform into the datasheet although all AI responses (n=480) were manually verified and recorded in the digital datasheet. The aggregate dataset comprised 1920 responses (AI systems: n=480; residents: n=1440). The complete response dataset and verification protocols are available in Supplemental Document 3 (available online at https://www.mcpdigitalhealth.org/): clinical decision-making response database.

### Outcomes Measures

#### Primary Outcome

The primary outcome measure was defined as diagnostic accuracy, calculated as the percentage of correct responses across all clinical scenarios. This enabled quantitative comparison between AI systems (n=480 responses) and residents (n=1440 responses).

#### Secondary Outcomes

We evaluated 8 key domains of AI implementation in OB-GYN decision-making, focusing on performance metrics, clinical integration potential, and system reliability. Secondary end points examined specific aspects of human-AI interaction in clinical scenarios.

1. AI performance stratification: A 3-tier classification system assessed diagnostic accuracy across the different AI platforms.
2. Resident expertise development: progression of clinical proficiency and decision-making capabilities across 5 residency years.
3. Linguistic impact assessment: performance in English and Italian scenarios was compared in AI systems and human residents.
4. Temporal pressure effects: diagnostic accuracy under time constraints was assessed to quantify the effect of stress on both AI systems and human practitioners.
5. Complexity assessment: standardized readability metrics were used to evaluate how linguistic complexity influences diagnostic performance.
6. Error pattern analysis: identification and comparison of error patterns in both high-performing AI systems and residents.
7. Integration potential assessment: the benefits and risks of AI integration were assessed at different stages of clinical expertise, highlighting feasibility in OB-GYN practice.
8. Response consistency: evaluation of response consistency between LLMs and residents across different languages and time constrictions.

### Statistical and Computational Methodology

Primary outcome analysis utilized 2-proportion Z-tests with Wilson confidence intervals. Secondary end points employed end point-specific statistical methodologies, including χ2 analysis for AI performance stratification, ANOVA for resident progression assessment, and multiple specialized tests for linguistic impact, temporal effects, and integration potential evaluation. All analyses were conducted using Python (version 3.11.6) within standardized Jupyter environments, employing established scientific computing libraries including pandas (2.2.3), numpy (1.24.4), and scipy (1.11.3) for statistical computations. Details are provided in Supplemental Document 4 (available online at https://www.mcpdigitalhealth.org/): technical framework and statistical analyses protocol for AI integration in OB-GYN decision-making.

## Results

### Primary Outcome: Overall Performance Comparison

**Figure 1:**
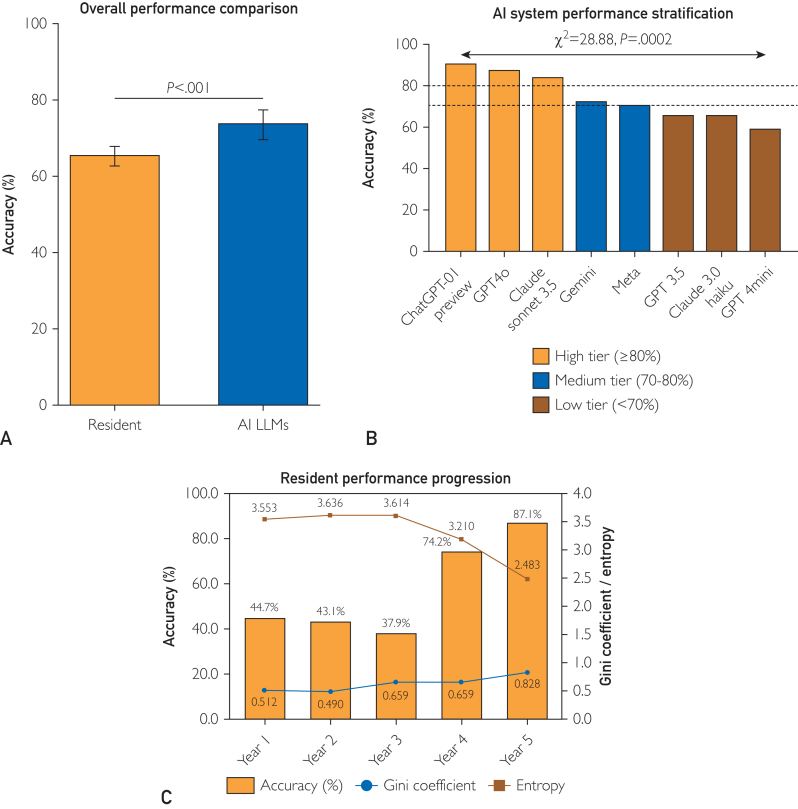
(A) Overall performance comparison: AI LLMs achieved an overall accuracy of 73.75% (95% CI, 69.64%-77.49%), exceeding resident performance 65.35% (95% CI, 62.85%-67.76%; *P*<.001) with an odds ratio (OR) of 1.49 and zero dropouts. This 8.40%-point difference corresponds to a moderate effect size (Cohen’s d=0.18). (B) AI system performance stratification: significant inter-platform variability (χ^2^=28.88; *P*=.0002) revealed 3 accuracy tiers among AI LLMs: high (≥80%; ChatGPT-01 preview 90.0%, GPT4o 86.7%, Claude Sonnet 3.5 83.3%), medium (70%-80%; Gemini 71.7%, and Meta 70.0%), and low (<70%; GPT 3.5 65.0%, Claude 3.0 Haiku 65.0%, GPT 4mini 58.3%). The highest-performing model (ChatGPT-01 preview) outperformed the lowest by a factor of 6.4 (*P*=.0001). (C) Resident performance progression: accuracy rates followed a non-linear trend: 44.7% in the first year (Gini=0.512, Entropy=3.553), 43.1% in the second year (Gini=0.490, Entropy=3.636), a dip to 37.9% in the third year (Gini=0.659, Entropy=3.614), and subsequent increases in the fourth (74.2%, Gini=0.659, Entropy=3.210), and fifth years (87.1%, Gini=0.828, Entropy=2.483).

The AI LLMs achieved an overall accuracy of 73.75% (95% CI, 69.64%-77.49%), significantly exceeding the resident performance of 65.35% (95% CI, 62.85%-67.76%; *P*<.001) with a 1·49 OR and zero dropout rates observed (Figure 1A). This performance differential of 8.40% points translated to a moderate effect size (Cohen’s d=0.18).

### Secondary Outcomes

The AI system performance stratification: Distinct performance tiers were evidenced among the evaluated AI LLMs, with significant inter-platform variations (χ^2^=28,88; *P*=.0002). The high-performance tier (≥80% accuracy) comprised ChatGPT-01 preview (90.0%), GPT4o (86.7%), and Claude Sonnet 3.5 (83.3%). The medium-performance tier (70%-80% accuracy) included Gemini (71.7%) and Meta (70.0%), whereas the lower-performance tier (<70%) consisted of GPT 3.5 (65.0%), Claude 3.0 Haiku (65.0%), and GPT 4mini (58.3%). Notably, the highest-performing system (ChatGPT-01 preview) reported 6.4-fold superior accuracy compared with the lowest-performing platform (*P*=.0001) (Figure 1B).

#### Resident Performance Progression

Year-wise accuracy rates found a non-linear progression pattern, with initial performance metrics of 44.7% for first-year residents (Gini=0.512, Entropy=3.553) and 43.1% for second-year residents (Gini=0.490, Entropy=3.636). A notable inflection point occurred during the third year of training, in which the accuracy temporarily decreased to 37.9% (Gini=0.659, Entropy=3.614), followed by substantial improvements in the fourth year (74.2%, Gini=0.659, Entropy=3.210) and fifth year (87.1%, Gini=0.828, Entropy=2.483) (Figure 1C).

#### Language Impact

The AI LLMs achieved 88.89% accuracy in English and 84.44% in Italian scenarios (differential: 4.45%). Resident performance reached 69.72% in English and 60.97% in Italian scenarios (differential: 8.75%). Between-group analysis revealed significant differences in both English (*P*=.02933) and Italian (*P*=.01662) domains. The AI LLMs systems reported a mean language impact of 15.83% (±12.22%), whereas residents reported 8.75% (±10.79%) impact. Between-group comparison yielded t=1.5044; *P*=.1429. High-performing AI LLMs exhibited 6.67% (±0.00%) language impact with 88.33% overall accuracy. Mid-to-low performing LLMs reported 18.89% (±13.93%) impact with 68.89% accuracy. Between-tier differential reached 12.22% (*P*=.284). High-performing residents reported 8.75% (±6.16%) language impact with 81.88% accuracy. Mid-to-low performing residents reported 11.67% (±10.26%) impact with 57.08% accuracy. Between-tier differential measured 2.92% (*P*=.470).

#### Time Pressure Effects on Clinical Decision-Making

**Figure 2:**
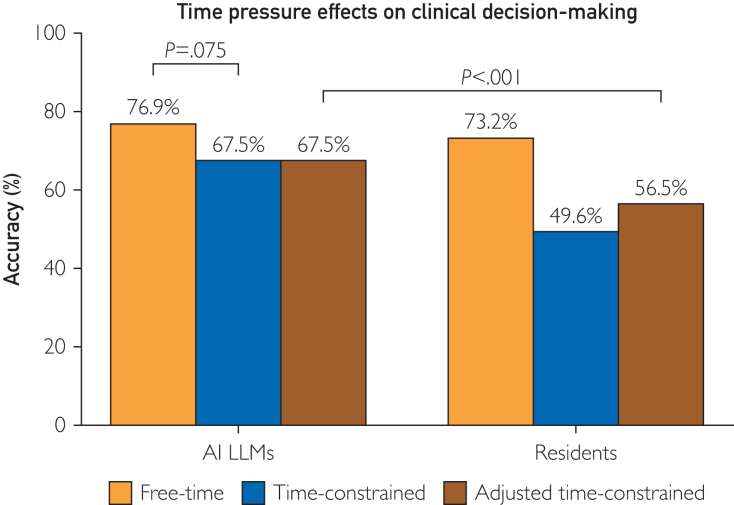
Time pressure effects on clinical decision-making. AI LLMs achieved 76.9% accuracy without time constraints and 67.5% under time pressure (9.4% differential, Cohen’s d=0.66; *P*=.075). In contrast, resident performance declined from 73.2% to 49.6% (23.6% differential, Cohen’s d=1.55; *P*<.001), a 14.3% greater deterioration compared with AI LLMs (Mann-Whitney U test; *P*=.009). When adjusted for inherent question difficulty (factor 1.139), resident accuracy decreased to 56.5% under time constraints, representing a 22.9% drop (Cohen’s d=1.009, Wilcoxon signed-rank test; *P*<.001, n=24).

The AI LLMs achieved 76.9% under free-time conditions and 67.5% under time constraints, with a 9.4% differential (Cohen's d=0.66; *P*=.075). Resident performance had an accuracy rate of 73.2% in free-time conditions declining to 49.6% under time constraints, yielding a 23.6% differential (Cohen’s d=1.55; *P*<.001). Between-group comparison reported a 14.3% greater performance deterioration among residents (Mann-Whitney U test; *P*=.009). Difficulty-adjusted analysis, implementing an inherent difficulty factor of 1.139, revealed an adjusted resident performance decline to 56.5% under time constraints, representing a 22.9% decrease (Cohen’s d=1.009, Wilcoxon signed-rank test; *P*<.001, n=24) (Figure 2).

#### Complexity Impact on Diagnostic Performance

Human residents exhibited negligible correlation between readability scores and accuracy rates (r=−0.0008), suggesting that clinical decision-making capabilities remained stable across varying levels of linguistic complexity. In contrast, AI LLMs reported a weak positive correlation with readability metrics (r=0.128), indicating modest sensitivity to linguistic complexity. Despite this correlation, the AI LLMs maintained superior overall diagnostic accuracy (73.75%, SD=0.295) compared with human residents (65.35%, SD=0.313). This performance differential persisted across the complexity spectrum, suggesting robust AI processing capabilities independent of linguistic variation. Detailed complexity score assessment processes for these scenarios are provided in Supplemental Document 5 (available online at https://www.mcpdigitalhealth.org/): complexity score assessment for GYN-OB questions.

#### Error Pattern Analysis

**Figure 3:**
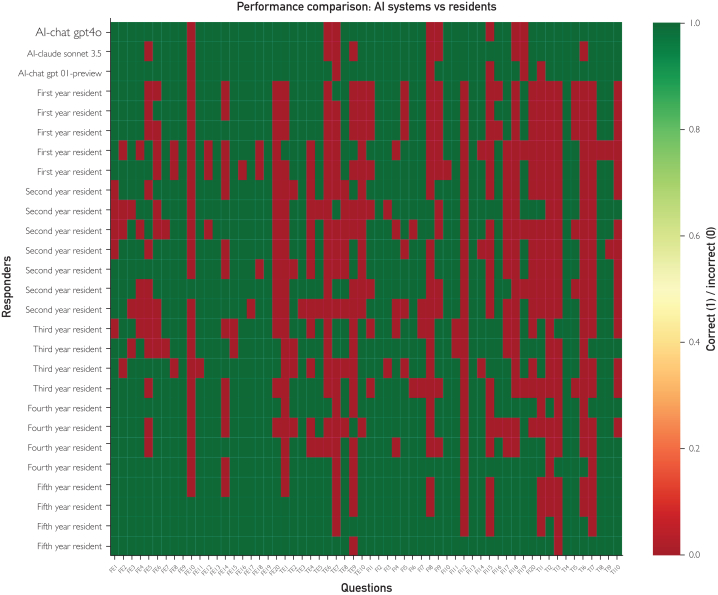
Performance comparison heatmap of AI systems vs residents. This heatmap displays question-by-question performance for 3 high-performing AI language models (rows 1-3) and 24 obstetrics-gynecology residents (subsequent rows), grouped by year of training (first through fifth). Columns represent the 60 multiple-choice scenarios—coded (eg, FE1, FI5, and TE2) to denote language (English/Italian) and time constraints (freely timed/time constrained). The color scale transitions from green (correct answer, value=1.0) to red (incorrect answer, value=0.0), illustrating individual-level accuracy for each question. The density of green cells in the top rows highlights the generally higher performance of AI systems, whereas shifts in color patterns among resident rows reflect varying accuracy by training level and scenario type. This visualization provides an at-a-glance comparison of overall performance, error distribution, and consistency across AI and human participants under different linguistic and temporal testing conditions.

**Figure 4:**
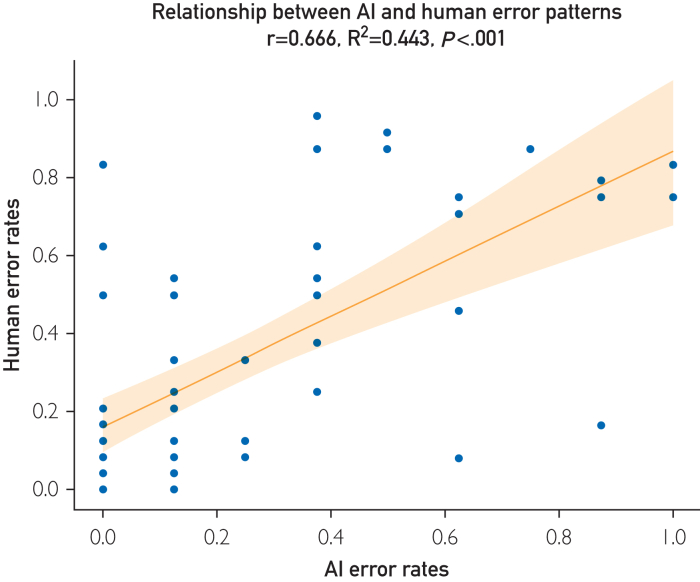
Correlation and performance stratification. This figure illustrates the relationship between AI and human error patterns, alongside performance stratification by resident training year. A scatter plot displays the correlation between errors made by high-performing AI models and residents (r=0.666; *P*<.001), with a fitted regression line (R^2^=0.443).

The top-performing AI LLMs chosen for the analysis, ChatGPT-01 preview, GPT4o, and Claude Sonnet 3.5, exhibited an error rate of 26.25%, compared with 34.65% for human residents. Error patterns from AI LLMs and residents are different (Figure 3). Correlation analysis revealed a moderately strong positive relationship between AI and human error patterns (r=0.666; *P*=6.43e-09), with regression analysis yielding an R^2^ value of 0.443. Performance stratification across training levels (mean accuracy=0.883, SD=0.321) indicated that first-year residents had substantial performance differentials relative to top AI systems (*P*<0.001, Cohen’s d=0.789); second-year residents maintained similar disparities (*P*<0.001, Cohen’s d=0.753); third-year residents reported moderate differences (*P*<0.001, Cohen’s d=0.638). Fourth-year residents reported diminishing but significant gaps compared with leading AI LLMs (*P*=0.0018, Cohen’s d=0.369), whereas fifth-year residents achieved performance parity with top-tier AI LLMs (*P*=0.736, Cohen’s d=0.038) (Figure 4).

#### Integration Potential Analysis

**Figure 5:**
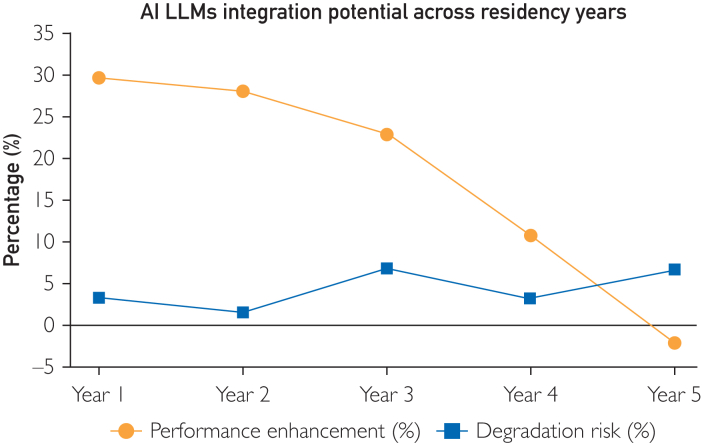
Integration potential analysis across residency years. This line chart compares performance enhancement (blue circles) and degradation risk (red squares) when AI LLMs assist residents from year 1 to year 5. First-year residents exhibit the greatest net benefit (+29.7%; *P*<.001, Cohen’s d=0.603) with minimal risk (3.3%), and second-year residents show similar gains (+28.1%; *P*<.001, and d=0.537) and a lower risk (1.7%). Third-year residents retain moderate improvement (+22.9%; *P*<.001, d=0.423) but face an elevated risk (6.7%; *P*<.05). Fourth-year gains diminish (+10.8%, *P*<.001, d=0.230) with a moderate risk (3.3%), and by the fifth year there is negligible or negative improvement (–2.1%; *P*=.447, d=–0.049) coupled with higher risk (6.7%; *P*<.05). These findings illustrate how the benefits of AI support decline and the risks increase as residents advance in training.

First-year residents reported substantial performance enhancement with AI LLMs integration (+29.7%; *P*<.001, Cohen’s d=0.603) and minimal degradation risk (3.3%). Second-year residents had comparable improvements (+28.1%; *P*<.001, Cohen’s d=0.537) with a lower degradation rate (1.7%); no significant differential was observed between these years (*P*=0.684). Third-year residents exhibited moderate enhancement (+22.9%; *P*<0.001, Cohen’s d=0.423) but increased degradation risk (6.7%; *P*<.05). Fourth-year residents maintained positive but diminishing benefits (+10.8%; *P*<0.001, Cohen’s d=0.230) with moderate degradation risk (3.3%), whereas fifth-year residents reported negligible improvement (–2.1%; *P*=0.447, Cohen’s d=–0.049) and elevated degradation risk (6.7%; *P*<.05) (Figure 5).

#### Cognitive Stability Assessment Results

Among the AI platforms, AI-chatGPT 01-preview, AI-chatGPT 3.5, and AI-chatGPT4O maintained 100% concordance in their answers across both languages and under both temporal conditions. In contrast, other AI systems displayed notable variability, with a 20% concordance deficit in English and a 40% deficit in Italian. Human participants also reported distinct patterns. First-year and second-year residents reported 100% concordance across all conditions and both languages. By the third year, English concordance dropped to 50%, whereas Italian remained at 100%. Fourth-year and fifth-year residents restored 100% concordance under all conditions. Introducing AI augmentation for third-year residents increased English concordance by 16.7%, although this improvement did not achieve statistical significance.

## Discussion

The integration of AI in OB-GYN decision-making represents a transformative step in the digital evolution of clinical practice and medical education. AI, broadly defined as technology enabling machines to simulate human cognitive functions like learning and decision-making,^16^ encompasses various subsets, such as machine learning, deep learning (DL), and generative AI. Machine learning involves training algorithms to make predictions on the basis of data, whereas DL uses multilayered neural networks to approximate complex human thought processes. The LLMs, like the ones used in our study, are advanced DL systems trained on extensive datasets to perform a wide range of language tasks. Our analyses reveal that certain AI LLMs not only surpassed residents in diagnostic accuracy (73.75% vs 65.35%; *P*<.001) but also maintained remarkable consistency under challenging conditions. Our findings provide new insights into AI’s performance in more complex, real-world clinical scenarios.^6^^,^^8^ Moreover, we examined how both time pressure and linguistic variability affected diagnostic reasoning, revealing that advanced AI LLMs and human residents respond differently under these stressors.

Significant variability emerged among AI platforms (χ^2^=28.88; *P*=.0002), with 3 top-performing models, which are ChatGPT-01 preview (90·0%), GPT4o (86·7%), and Claude Sonnet 3.5 (83.3%), consistently reporting minimal performance fluctuations across languages (6.67%±0.00%) and achieving high overall accuracy (88.33%). In contrast, lower-performing AI LLMs displayed greater linguistic variability (18.89%±13.93%) and diminished accuracy (68.89%). The superior cognitive stability and linguistic resilience of the high-performing models indicate that AI LLMs can potentially standardize care delivery across linguistic and cultural boundaries,^8^ an increasingly critical goal in diverse health care systems.

A crucial component of our analysis involved assessing decision-making consistency when identical questions were presented twice, once without time pressure and once under time pressure, in both English and Italian. The top 3 LLMs displayed 100% concordance in every scenario, reporting that their reasoning remained stable, unaffected by changing conditions. In contrast, human residents varied their responses under time pressure, illustrating how human judgment can be influenced by contextual stressors. This variability is not merely a curiosity but a potential challenge in high-stakes clinical environments in which inconsistent decisions can impact patient outcomes. The value of LLMs in this context is not that it explains why humans vary, but that it provides a reference point of unwavering consistency. Particularly when temporal constraints are intense, LLMs may serve as stabilizing agents, helping to ensure reliable decision-making. Such support could mitigate cognitive load and reduce the risk of variability in critical moments, complementing human expertise rather than replacing it.

Residents improve steadily with training, reporting an ∼12·0% annual growth in diagnostic accuracy and evidence of increasing decision-making sophistication (decreasing entropy and increasing Gini coefficients). Despite this growth, a correlation in error patterns between top-performing LLMs and residents (r=0.666; *P*=6.43e-09; R^2^=0.443) suggests shared underlying cognitive frameworks. Even as residents develop more nuanced skills, LLMs can complement human reasoning, particularly in complex scenarios in which cognitive load and linguistic factors come into play.

Artificial intelligence augmentation impacts clinical performance differently across residency levels. In the early years (1-2), residents achieved substantial performance gains (29.7%, 28.1%; both *P*<.001) with minimal risks, suggesting that LLMs can solidify foundational skills. By the third year, when trainees transition toward greater autonomy, benefits persisted (22.9%; *P*<.001) but were accompanied by a higher risk of performance degradation (6.7%; *P*<.05), indicating the need for careful, context-specific integration. In the later stages (years 4-5), improvements diminished (10.8% in year 4; −2.1% in year 5), and degradation risks rose (3.3%-6.7%), highlighting that as residents approach full independence, AI support may conflict with their established reasoning strategies and thus requires more selective application. This evolving landscape suggests that one-size-fits-all integration approaches may be inadequate. Instead, tailored strategies that consider the trainee’s skill level, clinical complexity, and the nature of the LLMs’ support are necessary.

Despite the promising capabilities of LLMs, it is essential to acknowledge limitations and potential risks. AI models, including LLMs, can generate inaccurate or misleading information, so-called hallucinations, which may not be immediately apparent due to the models’ high linguistic proficiency.^4^^,^^17^ This underscores the necessity for vigilant human oversight and expert validation of AI-generated outputs, especially in clinical settings where errors can have important consequences. Moreover, AI models may harbor inherent biases stemming from their training data, which may not accurately represent diverse patient populations.^6^^,^^18^, ^19^, ^20^, ^21^ These biases can lead to unequal performance across different demographic characteristic groups, potentially exacerbating health care disparities. Explainable AI (XAI) is another crucial aspect, referring to AI systems designed to make their decision-making processes transparent and interpretable.^22^^,^^23^ As LLMs grow more complex, understanding how they arrive at specific conclusions becomes more challenging, yet it is vital for building trust and facilitating proper integration into clinical decision-making. Enhancing XAI methods will help clinicians interpret AI recommendations, fostering a collaborative environment in which AI augments human expertise effectively.

Our findings suggest that the integration of LLMs into OB-GYN training requires stage-specific approaches. Early in the training process, incorporating AI tools directly into curricula can bolster foundational clinical reasoning skills. During the critical third-year transition, it may be necessary to implement tailored protocols to ensure that AI reinforces rather than undermines the resident’s growing autonomy. At more advanced stages, selective and judicious use of LLMs can complement refined clinical reasoning, mitigating the risk of performance degradation. Although the overall degradation rate is low (4.3%) and top-tier AI systems report stable performance, the risks observed among advanced trainees reinforce the importance of context-aware integration. Continued research is needed to refine these frameworks, address ethical concerns such as biases and explainability, and ensure that AI augments clinical practice rather than hinders it. We recognize certain limitations and suggest future directions for LLM-based evaluation. First of all, ensuring that none of our examination questions appeared in the LLMs’ training corpus is inherently challenging, even though we introduced extra distractors and unorthodox answer choices to reduce the risk of overlap. Second, determining whether routine AI assistance accelerates or impedes the development of independent diagnostic reasoning will require further investigation. Third, although some fifth-year residents in our study matched or exceeded the accuracy of top-performing LLMs, how these models compare to fully practicing physicians or subspecialists—who may have deeper but more narrowly focused expertise—remains unknown. Finally, there is a need to establish standards for evaluating and validating AI tools in health care, similar to the rigorous processes used for drugs and medical devices.^17^^,^^24^, ^25^, ^26^ Developing clear guidelines will facilitate responsible deployment, ensuring that AI interventions are safe, effective, and aligned with patient-centered care.

## Conclusion

Our findings indicate that high-performing AI language models have the potential to augment clinical reasoning in OB-GYN, offering benefits in accuracy, consistency, and resilience, especially under pressure. However, responsible integration requires matching AI tools to trainee experience, upholding ethical safeguards, and maintaining human oversight. By leveraging AI’s strengths and mitigating its limitations, we can improve both education and patient care. Future efforts should refine integration models, emphasize equitable and explainable AI use, and adapt to ongoing technological advances.

## Potential Competing Interests

The authors report no competing interests.

## Funding

This study did not receive any funding or financial support.

## Ethics Statement

This study was conducted at the School of Obstetrics and Gynecology, University of Messina (Italy), under the academic supervision of its faculty board and learning purpose for the resident and entirely voluntary. The research design involved a purely observational survey using multiple-choice questions; no investigational drugs, medical devices, or clinical interventions were employed. In accordance with Italian legislation—specifically the Italian Data Protection Code (D.Lgs. 196/2003 as updated by D.Lgs. 101/2018, which exempts anonymized data from general data protection regulation obligations) formal ethics committee review was not required. All data were fully anonymized at the point of collection, and no personally identifiable information was recorded. All participants provided written informed consent before enrolling in this study. The full text of the consent form is reported in Supplemental Document 6 (available online at https://www.mcpdigitalhealth.org/): “SD6-Informed Consent”.

## Supplemental Online Material

Supplementary Document S5

Supplementary Document SD6
