# Supplementary material for: The PERFORM Study: Artificial Intelligence Versus Human Residents in Cross-Sectional Obstetrics-Gynecology Scenarios Across Languages and Time Constraints
Source: Mayo Clin Proc Digit Health. 2025 Mar 8;3(2):100206. doi: 10.1016/j.mcpdig.2025.100206 (PMC12190988; doi:10.1016/j.mcpdig.2025.100206)
Supplement: Supplementary document 1 [file mmc1.docx]

**Supplementary document 1 “SD1: clinical case scenario and STROBE guideline”**

This supplementary document presents a comprehensive compilation of gynecological-obstetric clinical scenarios, accompanied by detailed adherence to STROBE (Strengthening the Reporting of Observational Studies in Epidemiology) guidelines. The clinical scenarios were systematically categorized into four distinct assessment domains, reflecting variations in both temporal constraints and linguistic parameters.

The validation of correct responses was established through reference to authoritative sources in obstetrics and gynecology, including the American College of Obstetricians and Gynecologists (ACOG) examination materials, the Italian Ministry of University and Research (Ministero dell'Università e Ricerca) specialty entrance examination protocols, and the widely-recognized CASE FILES® OBSTETRICS & GYNECOLOGY (Third Edition, Toy, Baker, Ross, & Jennings). This academic resource is particularly notable for its structured approach to clinical problem-solving, featuring 60 comprehensive obstetrics and gynecology cases designed to foster analytical thinking rather than rote memorization, complemented by USMLE-format review questions and valuable clinical insights.

The classification system yielded a structured taxonomy comprising 60 total scenarios, distributed across four primary categories: freely timed English scenarios (FE, n=20), freely timed Italian scenarios (FI, n=20), time-constrained English scenarios (TE, n=10), and time-constrained Italian scenarios (TI, n=10). To enhance the diagnostic challenge and evaluate advanced clinical reasoning capabilities, selected scenarios were augmented with additional plausible but incorrect response options, specifically designed to test the discriminative abilities of both artificial intelligence language models and human practitioners.

Below is the original text with standardized formatting for the multiple-choice questions. The wording and punctuation remain unchanged. Only the layout has been adjusted for clarity, ensuring each question and its options are clearly listed. Each question and set of answer choices is separated, and the multiple-choice options are each on a new line.

**FE1.**
A 48-year-old G3 P3 woman complains of a 2-year history of loss of urine four to five times each day, typically occurring with coughing, sneezing, or lifting; she denies dysuria or the urge to void during these episodes. These events cause her embarrassment and interfere with her daily activities. The patient is otherwise in good health. A urine culture performed 1 month previously was negative. On examination, she is slightly obese. Her blood pressure is 130/80 mm Hg, her heart rate is 80 beats per minute, and her temperature 99°F (37.2°C). The breast examination is normal without masses. Her heart has a regular rate and rhythm without murmurs. The abdominal examination reveals no masses or tenderness. A midstream voided urinalysis is unremarkable.

Most likely diagnosis:
a. Genuine stress incontinence.
b. mixed urinary incontinence
c. Urge incontinence
d. Urinary tract infection
e. Bladder cancer

**FE2.**
Referring to the previous clinical case, which physical examination would you find?:

a. Hypermobileurethra, cystocele, or loss of urethrovesical angle.
b. Hypomobileurethra, rectocele
d. Dysuria and hematuria
e. hematochezia

**FE3.**
A 66-year-old woman comes in for a routine physical examination. Her menopause occurred at age 51 years, and she is currently taking an estro- gen pill along with a progestin pill each day. The past medical history is unremarkable. Her family history includes one maternal cousin with ovarian cancer. On examination, she is found to have a blood pressure of 120/70 mm Hg, heart rate of 70 beats per minute, and temperature of 98°F (36.6°C). She weighs 140 lb and is 5 ft 4 in tall. The thyroid is normal to palpation. Examination of her breasts reveals no masses or discharge. The abdominal, cardiac, and lung evaluations are within normal limits. The pelvic examination shows a normal, multiparous cervix, a normal-sized uterus, and no adnexal masses. She had undergone mammography 3 months previously.

What is not indicated as next steps?
a. pet
b. Stool for occult blood,
c. colonoscopy or barium enema/flexible sigmoidoscopy,
d. pneumococcal vaccine, influenza vaccine, tetanus vaccine (if not within 10 years), cholesterol screening,
e. fasting blood sugar,
f. thyroid function tests,
g. bone mineral density screening, and urinalysis.

**FE4.**
Referring to the previous clinical case, What would be the most common cause of mortality for this patient?
a. Cardiovasculardisease
b. breast cancer
c. Neurological diseases
d. lung cancer

**FE5.**
An 18-year-old adolescent female is being seen for a health maintenance appointment. She has not had a Pap smear previously. She currently takes oral contraceptive pills. She began sexual intercourse 1 year previously.

Which of the following statements is most accurate regarding health maintenance for this individual?
1.A Pap smear should not be performed in this patient at this time.
2.The HPV vaccine should be administered only if she has a history of genital warts.
3.The most common cause of mortality for this patient would be suicide.
4.Hepatitis C vaccination should be offered to this patient.

**FE6.**
After a 4-hour labor, a 31-year-old G4 P3 woman undergoes an uneventful vaginal delivery of a 7 lb 8 oz infant over an intact perineum. During her labor, she is noted to have mild variable decelerations and accelerations that increase 20 beats per minute (bpm) above the baseline heart rate. At delivery, the male baby has Apgar scores of 8 at 1 minute, and 9 at 5 minute. Slight lengthening of the cord occurs after 28 minute along with a small gush of blood per vagina. As the placenta is being delivered, a shaggy, reddish, bulging mass is noted at the introitus around the placenta.

What is the most likely diagnosis?
A. Uterine inversion.
B. residual placenta
C. vaginal edema
D. second unknown fetus

**FE7.**
Referring to the previous clinical case, What is the most likely complication to occur in this patient?
A. Post partum hemorrhage.
B. MYOCARDIAL INFARCTION
C. CID
D. TVP

**FE8.**
A 32-year-old G1 P0 woman at 40 weeks’ gestation undergoes a normal vaginal delivery. Delivery of the placenta is complicated by an inverted uterus, with subsequent hemorrhage leading to 1500 mL of blood loss. She is managed with a transfusion of erythrocytes.

Which of the following is the best explanation of the mechanism of hemorrhage?
1.Inverted uterus stretches the uterus,causing trauma to blood vessels leading to bleeding.
2.Inverted uterus leads to inability for an adequate myometrial contraction effect.
3.Inverted uterus causes a local coagulopathic reaction to the uterus and endometrium.
4.Inverted uterus causes muscular abrasions and lacerations leading to bleeding.

**FE9.**
A 49-year-old woman complains of irregular menses over the past 6 months, feelings of inadequacy, vaginal dryness, difficulty sleeping, and episodes of warmth and sweating at night. On examination, her blood pressure is 120/68 mm Hg, heart rate is 90 beats per minute, and temperature is 99°F (37.2°C). The cardiac and lung examinations are unremarkable. The breasts are symmetric, without masses or discharge. Examination of the external genitalia does not reveal any masses.

What is the most likely diagnosis?
1 Lupus
2 AIDS
3 Lymphoma
4 DM
5 Climacteric syndrome

**FE10.**
Referring to the previous clinical case, what is not necessary as Next diagnostic step:
a. Serumfollicle-stimulatinghormone(FSH)
b. luteinizing hormone (LH).
c. Tsh plasmatic level
d. Tyroid US
e. Pelvic US

**FE11.**
A 57-year-old woman comes to the physician 1 week after noticing a mass in her left breast during breast self-examination. Menopause occurred 6 months ago. She was receiving estrogen therapy but discontinued it 6 weeks ago; she has had no menopausal symptoms. There is no family history of breast cancer. Examination shows a 2-cm, palpable, nontender, mobile mass in the upper outer quadrant of the left breast; no nipple discharge can be expressed. Examination of the right breast shows no abnormalities.

Which of the following is the most appropriate next step in management?
(A) Reexamination in 3 months
(B) Mammography
(C) CT scan of the chest
(D) Ductal lavage
(E) Mastectomy

**FE12.**
A 27-year-old nulligravid woman has had severe pain with menses that has caused her to miss at least 2 days of work during each menstrual cycle for the past year. She has occasional pain during sexual intercourse. She weighs 50 kg (110 lb) and is 160 cm (5 ft 3 in) tall; BMI is 20 kg/m2. Pelvic examination shows a normal-appearing vulva and vagina. The cervix is pink with minimal endocervical gland eversion. The uterus is normal in size. The left ovary is 2 × 3 cm; the right is 4 × 6 cm

Which of the following is the most likely cause of her condition?
(A) Chronic appendicitis
(B) Endometriosis
(C) Pelvic congestion syndrome
(D) Polycystic ovarian syndrome
(E) Premenstrual syndrome

**FE13.**
A 42-year-old woman, gravida 3, para 3, comes to the physician because she has not had a menstrual period for 2 months. She reports that she had an episode of spotting 3 weeks ago. She has had no other symptoms. She has no history of abnormal Pap smears; her last Pap smear was 10 months ago. She is sexually active with her husband and uses condoms. She is 163 cm (5 ft 4 in) tall and weighs 72 kg (160 lb); BMI is 28 kg/m2. On physical examination, the abdomen is nontender to palpation. Pelvic examination shows a slightly enlarged uterus; there are no palpable adnexal masses.

Which of the following is the most appropriate next step in management?
(A) Measurement of serum β-hCG concentration
(B) Measurement of serum thyroid-stimulating hormone concentration
(C) CT scan of the pelvis
(D) Oral contraceptive therapy
(E) Endometrial biopsy

**FE14.**
A 15-year-old girl is brought to the physician by her mother because she believes that her daughter has become sexually active and wants her to use contraception. During an interview with the patient alone, she reports that she has become sexually active with one male partner over the past 3 months. She and her partner use condoms consistently, and she is not interested in any other form of contraception at this time. Menarche was at the age of 12 years, and menses occur at regular 28-day intervals. She has had no symptoms of sexually transmitted diseases. Examination shows no abnormalities.

In addition to counseling the patient about all contraceptive methods, which of the following is the most appropriate next step?
(A) Do not inform the mother that her daughter and her partner use condoms, but encourage the daughter to communicate the information
(B) Inform the mother that her daughter and her partner use condoms
(C) Inform the mother that her daughter is protecting herself appropriately against pregnancy, but do not mention what form of contraception
(D) Inform the mother that you have administered depot medroxyprogesterone to the patient
(E) Inform the mother that you have given the patient a prescription for an oral contraceptive

**FE15.**
Two hours after vaginal delivery at term of a 3062-g (6-lb 12-oz) newborn, a 32-year-old woman, gravida 3, para 3, has the onset of heavy vaginal bleeding. Labor was augmented with oxytocin because of a prolonged first stage and required forceps delivery over a midline second-degree episiotomy. The abdomen is soft and nontender. Examination shows a boggy uterus palpated 4 cm above the umbilicus. The perineum is intact.

Which of the following is the most likely cause of this patient’s hemorrhage?
(A) Disseminated intravascular coagulation
(B) Episiotomy site bleeding
(C) Uterine atony
(D) Uterine infection
(E) Uterine rupture

**FE16.**
A 19-year-old primigravid woman at 8 weeks’ gestation is brought to the emergency department because of light vaginal bleeding and mild lower abdominal cramps during the past 8 hours. Her temperature is 37°C (98.6°F), pulse is 84/min, respirations are 18/min, and blood pressure is 110/70 mm Hg. Abdominal examination shows no tenderness or masses; bowel sounds are normal. On pelvic examination, there is old blood in the vaginal vault and at the closed cervical os. The uterus is consistent in size with a 6- to 8-week gestation. Transvaginal ultrasonography shows an intrauterine pregnancy. A fetal heartbeat is seen.

Which of the following is the most appropriate next step in management?
1.Discharge home for observation
2.Oral administration of misoprostol
3.Intramuscular administration of methotrexate
4.Operative laparoscopy
5.Dilatation and curettage

**FE17.**
A 16-year-old girl is brought to the emergency department 6 hours after the onset of moderate lower abdominal cramps and intermittent nausea. She has not vomited during this time. She says that her last menstrual period was 2 months ago, but she has had intermittent bleeding since then, including spotting for the past 2 days. Menarche was at the age of 15 years. Menses occur at irregular 25- to 45-day intervals. She is sexually active and uses condoms inconsistently. Her temperature is 38.1°C (100.6°F), pulse is 94/min, respirations are 22/min, and blood pressure is 120/80 mm Hg. Examination shows a soft abdomen with lower quadrant tenderness, especially on the right. Bowel sounds are normal. Pelvic examination shows scant vaginal bleeding and a palpable, tender right adnexal mass. The cervix appears normal. There is no cervical motion tenderness.

Which of the following is the most appropriate next step in management?

1. Complete blood count
2. Measurement of serum β-hCG concentration
3. Abdominal x-ray
4. Ceftriaxone and azithromycin therapy
5. Exploratory laparoscopy

**FE18.**
A 13-year-old girl is brought to the physician because of a 1-year history of intermittent irregular vaginal bleeding; the bleeding ranges from spotting to heavier than a normal menstrual period, occurs every 2 to 8 weeks, and lasts 10 to 30 days. Examination shows a pink, well-rugated vagina with no discharge; the cervix appears normal. The uterus is 6 cm in length, regular in contour, and nontender. There are palpable, normal-sized, nontender ovaries.

Which of the following is the most appropriate pharmacotherapy to alleviate this patient’s symptoms?
1.Continuous low-dose estrogen
2.Gonadotropin-releasing hormone agonist
3.Oral contraceptives
4.Tetracycline
5.Thyroid hormone

**FE19.**
A 22-year-old primigravid woman at 34 weeks’ gestation is brought to the emergency department by ambulance after being found unconscious by her husband. Paramedics report that she was having tonic-clonic movements that have now stopped. Her last visit to the physician was 2 weeks ago. Pregnancy had been uncomplicated. On arrival, she is awake, lethargic, and mildly confused. She says that she had a headache and did not feel well earlier in the day. She has no history of serious illness. Her only medication is a prenatal vitamin. Her temperature is 37°C (98.6°F), pulse is 80/min, respirations are 18/min, and blood pressure is 170/110 mm Hg. Examination shows a nontender, soft uterus consistent in size with a 34-week gestation. Cranial nerves are intact. Motor function is normal. Deep tendon reflexes are 3+.

Which of the following is the most likely diagnosis?
1.Absence seizures
2.Cerebral infarction
3.Eclampsia
4.Migraine
5.Pheochromocytoma

**FE20.**
A 38-year-old G2P2 is BRCA-1 positive. What is the best way for her to lower her risk of cancer?
A. Begin oral contraceptive pills
B. Begin tamoxifen daily
C. Perform BSO now
D. Plan hysterectomy and BSO at time of menopause
E. Schedule an MRI and Mammograms every 6 month

**TE1.**
A 32-year-old patient comes in complaining of nipple discharge. A light green nipple discharge is elicited on exam. No mass is palpated. What is the most likely diagnosis?

A. Breast abscess
B. Ductal carcinoma in situ
C. Ductal ectasia
D. Intraductal papilloma
E. Prolactinoma

**TE2.**
What genetic mutation has the highest risk of breast cancer?

A. BRCA-1
B. BRCA-2
C. FAP (Familial adenomatous polyposis)
D. HNPCC
E. Li Fraumeni

**TE3.**
Which tumor marker is characteristic of a granulosa cell tumor?

A. AFP
B. CA 19-9
C. CA 125
D. Inhibin
E. LDH

**TE4.**
Trisomy results most often as a result of nondisjunction during which phase of the cell cycle?

A. Maternal meiosis I
B. Maternal meiosis II
C. Mitosis
D. Paternal meiosis I

**TE5.**
What is the most likely cause of infertility in a couple with normal HSG and monthly menses?

A. anovulation
B. luteal phase defect
C. male factor
D. tubal factor

**TE6.**
22-year-old patient has an 8 cm myoma. Which is the least reason to operate?

A. Heavy menstrual bleeding
B. Infertility
C. Pelvic pain
D. Prolapsed fibroid

**TE7.**
A 33-year-old multigravida patient presents complaining of dyspareunia and “feeling loose” during intercourse. Obstetrical history includes that of an episiotomy with a 4th-degree extension during her last delivery several years ago. Review of Systems include rare symptoms of stress urinary incontinence, but no complaints of fecal incontinence. Pelvic examination is significant for a widened genital hiatus, scarred posterior fourchette, positive dovetail sign and a tender perineal body that is <0.3cm. The most likely reason that the patient has no symptoms of fecal incontinence is:

A. The external anal sphincter is mostly intact
B. The internal anal sphincter is mostly intact
C. The recto anal inhibitory reflex is normal
D. The anorectal angle is less acute
E. The dependency on the puborectalis muscle is increased

**TE8.**
A 17-year-old G0 presents to your office with secondary amenorrhea. She menstruated for 4 years, but her last menses was 11 months ago. History and physical exam are otherwise unremarkable. Laboratory testing reveals:

Prolactin 15 ng/mL (3 – 30)
TSH 2.1 mIU/L (0.4 – 4.5)
FSH 45 IU/L (follicular phase 2.5 – 10)
Estrogen 20 pg/mL (follicular phase 19 -144)
HCG < 5 mIU/mL (non-pregnant < 5)

What is the cause of this patient’s amenorrhea?

A. hyperprolactinemia
B. hypogonadotropic hypogonadism
C. Mullerian Agenesis
D. ovarian failure
E. subclinical hypothyroidism

**TE 9.**
Referring to the previous clinical case , Which of the following is a risk factor for endometrial ablation failure?

A. Age older than 40
B. History of dyspareunia
C. Parity greater than 5
D. Prior use of oral contraceptives

**TE10.**
A 38-year-old G2P2 is BRCA-1 positive. What is the best way for her to lower her risk of cancer?

A. Begin oral contraceptive pills
B. Begin tamoxifen daily
C. Perform BSO now
D. Plan hysterectomy and BSO at time of menopause
E. Schedule an MRI and Mammograms every 6 month

**FI1.**
Lo Sniff test individua la presenza di:

A: vaginosi batterica
B: infezione da Trichomonas
C: infezione da Clamidia
D: infezione da Micoplasma

**FI2.**
Il punteggio di Bishop valuta:

A: la possibilità di successo dell'induzione del travaglio
B: lo sviluppo dei caratteri sessuali secondari
C: la maturazione follicolare
D: il rischio di preeclampsia

**FI3.**
Che farmaco contengono gli IUD medicati?

A: Levonorgestrel
B: Desogestrel
C: Medrossiprogesterone acetato
D: Enantone

**FI4.**
Individuare l'affermazione NON corretta relativa alla PID (Malattia Infiammatoria Pelvica).

A: Raramente interessa la tuba
B: È più frequente nelle donne tra 20-30 anni
C: È collegata alla molteplicità dei partner sessuali
D: Uno degli agenti eziologici è la Chlamydia Trachomatis

**FI5.**
Ad una paziente di 30 anni, nullipara, viene posto il sospetto diagnostico di endometriosi pelvica. Quale dei seguenti trattamenti dell'endometriosi è ERRATO?

A: Estrogeni
B: Estroprogestinici
C: Danazolo
D: Progestinici

**FI6.**
Tutti i seguenti sintomi riportati dalla paziente sono coerenti con l'ipotesi diagnostica TRANNE uno. Quale?

A: Ipomenorrea
B: Dismenorrea
C: Dispareunia
D: Disuria

**FI7.**
Una donna di 50 anni si presenta chiedendo informazioni relative al papilloma virus umano (HPV) e alla vaccinazione che le hanno proposto per la figlia di 12 anni. In relazione alla vaccinazione, la paziente viene informata che esistono due tipi di vaccini: il bivalente e il quadrivalente, quale delle seguenti affermazioni è corretta?

A: entrambi i vaccini sono altamente immunogenici ed efficaci
B: uno viene somministrato con iniezione endovenosa e l'altro con iniezione sottocutanea
C: il bivalente richiede 2 somministrazioni e il quadrivalente 4 somministrazioni
D: la vaccinazione è assolutamente sicura anche in gravidanza

**FI8.**
Quale delle seguenti affermazioni relative a pap test e HPV è FALSA?

A: Un PAP test annuale tra i 20 e i 29 anni ha probabilità di non diagnosticare una lesione di circa il 5%
B: Un singolo PAP test ha una sensibilità dell'85% per HPV
C: Il PAP test ha una specificità per le lesioni indotte dal virus del 95%
D: Il PAP test consente di diagnosticare il 90% delle neoplasie cervicali se ripetuto ogni 3 anni

**FI9.**
Una donna di 35 anni ha una pressione sanguigna di 80/40 mm Hg, febbre e dolore addominale. Quale dei seguenti è il probabile meccanismo dell'ipotensione del paziente?

a.Disfunzione della contrattilità cardiaca
B. Bradicardia
C. Stravaso nel terzo spazio
D. Vasodilatazione

**FI10.**
Una ragazza di 16 anni presenta oligomenorrea e ipertricosi. Quale tra questi esami NON è indicato?

A: Ecografia epatica
B: Dosaggio degli androgeni e del rapporto LH/FSH
C: Dosaggio della prolattina
D: Ecografia pelvica

**FI11.**
Una paziente in occasione di una visita di controllo vi esprime perplessità relative al fatto che la figlia di 12 anni ha uno sviluppo fisico adeguato alla sua età (97° percentile, comparsa di peli e iniziale ingrossamento delle mammelle) ma non ha ancora avuto il menarca, a differenza di molte sue compagne di classe. La bambina è nata con parto eutocico dopo una gravidanza normodecorsa, è sana e ha una vita attiva adeguata alla sua età. Quale delle seguenti informazioni vi porta a considerare la diagnosi di imene imperforato?

A: Frequenti accessi in pronto soccorso per dolore addominale rimasti senza diagnosi
B: Consistente riduzione della massa grassa per veloce crescita in altezza
C: Perdita di abbondanti secrezioni mucose dalla vagina
D: Recente diagnosi di celiachia

**FI12.**
Una donna di 25 anni ha una storia di 1 anno di amenorrea a causa di iperprolattinemia. Ha una galattorrea bilaterale dovuta a un adenoma secernente prolattina. Quale dei seguenti test è in grado di rivelare un risultato anomalo?

1.Scansione DEXA della colonna vertebrale
2.Biopsia endometrio.
3.Mammografia del seno
4. Livello dell'ormone stimolante la tiroide (TSH)

**FI13.**
Una donna di 28 anni che ha subito un parto cesareo 1 settimana fa viene portata al pronto soccorso con una pressione arteriosa di 60/40 mm Hg. Il marito della paziente afferma che ha avuto 2 giorni di nausea e vomito, febbre a 38,8 ° C e mialgie. Il motivo del cesareo è stato l'arresto della fase attiva, con dilatazione cervicale a 5 cm per 3 ore nonostante le forti contrazioni uterine. È stata dimessa a casa il 3° giorno post-operatorio in buone condizioni. All'esame, il paziente appare letargico e ha confusione mentale. L'auscultazione del cuore rivela tachicardia. L'esame polmonare mostra lievi crepitii alle basi polmonari. L'addome è dolente dappertutto e il fondo dell'utero è leggermente dolente. L'incisione cutanea è tenera, rossa e indurita. All'apertura dell'incisione, viene estratto materiale purulento. Il tessuto sottostante è palpato e ha una consistenza muscolosa con crepitanza. La valutazione di laboratorio rivela un livello di emoglobina di 15 g/dL e una creatinina sierica di 2,1 mg/dL.

Qual è la diagnosi più probabile?
a.Fascite necrotizzante.
b. Encefalite
c.Sindrome paraneoplastica
d. diselettrolitemia

**FI14.**
Rispetto al caso clinico precendete, Qual è il prossimo passo nella terapia?

a.Isotonico fluidi endovenosi, antibiotici ad ampio spettro e debritment chirurgico immediato.
b.Consulenza chirurgica
c.Trasferimento in terapia intensiva
d.Terapia con cortisonici

**FI15.**
Una donna di 26 anni G1 P0 a 39 settimane di gestazione viene ricoverata in ospedale in travaglio. Si nota che ha contrazioni uterine ogni 7-10 minuti. La sua storia antepartum è significativa per uno stato di rosolia non immune. All'esame, la sua pressione sanguigna (BP) è di 110/70 mm Hg e la frequenza cardiaca (FC) è di 80 battiti al minuto (bpm). Il peso fetale stimato è di 7 libbre. All'esame pelvico, è stato notato un cambiamento negli esami cervicali da una dilatazione di 4 cm a 7 cm nelle ultime 2 ore. Il bacino viene valutato adeguato all'esame digitale.

Quali sarebbero scelte sbagliate? Indicare più di una
a.Continuare a osservare il travaglio.
b.Usare ossitocina
c.Taglio cesareo
d.Induzione di parto abortive

**FI16.**
Una femmina G0 P0 di 23 anni si presenta in ufficio lamentando cicli irregolari sin dal menarca. Dopo ulteriori domande, ha anche notato un aumento dei peli sul viso e dell'acne per molti anni. Nega qualsiasi storia di problemi medici e ha una forte storia medica familiare di diabete. All'esame, si nota che ha una pressione sanguigna (BP), polso, frequenza respiratoria e temperatura normali. È obesa con un indice di massa corporea (BMI) di 34. È nota per avere un po' di irsutismo e acanthosis nigricans (del collo e dell'interno coscia). Il suo esame pelvico è limitato dalla sua obesità ma normale. Non desidera una gravidanza in questo momento. Il suo test di gravidanza è negativo.

Qual è la diagnosi più probabile?
a.PCOS.
b.Cancro ovarico
c.Endometrioma
d.Adenoma surrenalico
e.Sindrome di Addison

**FI17.**
Quali complicanze è a rischio per il paziente? Scegli più di una
1.Diabete mellito,
2. cancro dell'endometrio,
3. iperlipidemia,
4. sindrome metabolica,
5.malattie cardiovascolari.

**FI18.**
Qual è il tuo prossimo passo diagnostico? Scegli più di uno:

1. TSH prolattina
2. deidroepiandrosterone solfato (DHEA-S),
3. 17-idrossiprogesterone,
4. ecografia pelvica.

**FI19.**
Qual è il tuo piano terapeutico da non scegliere per questa paziente? Scegli più di una
1.Regolare i cicli mestruali con contraccettivi orali combinati
2.screening per anomalie metaboliche (DM, pannello lipidico, ecc.).
3.Incoraggia la dieta e l'esercizio fisico.
4.Vieta l’attività sessuale
5.Drilling ovarico laparoscopico
6.Astensione assoluta da cioccolato al latte
7.Se la paziente è un medico dovrebbe astenersi dalla sala operatoria
8.Se la paziente è un pompiere non dovrebbe salire sulle scale

**FI20.**
Quale delle seguenti affermazioni su T pallidum è corretta?
1.È un protozoo.
2.Se si è infetti, è sconsigliato il concepimento
3.Provoca malattie neonatali principalmente per inoculazione di retta del bambino dalla lesione genitale.
4. I trattamenti alternativi includono la doxiciclina e l'eritromicina.

**TI1.**
Una donna di 31 anni G2 P1 a 40 settimane di gestazione è progredita nel travaglio da 5 cm a 6 cm di dilatazione cervicale nell'arco di 2 ore. Quale delle seguenti parole descrive meglio il travaglio?

1.Fase latente prolungata
2.Fase attiva prolungata
3.Arresto della fase attiva
4.Fase attiva prolungata
5.Travaglio normale

**TI2.**
Una donna G2 P1 di 24 anni a 39 settimane di gestazione si presenta con contrazioni uterine dolorose. Si lamenta anche di sangue vaginale scuro misto a muco. Quale delle seguenti affermazioni descrive l'eziologia più probabile del suo sanguinamento?

A)Placenta previa.
B) Distacco della placenta.
C) "bloody show".
D) Vasa previa .
E) lacerazione vaginale.

**TI3.**
Una donna di 32 anni G0 P0 è nota per avere mestruazioni irregolari e irsutismo. Quale dei seguenti è compatibile con la sindrome dell'ovaio policistico?

A. Livelli elevati di 17-idrossiprogesterone
B. Massa ovarica di 9 cm
C. Sanguinamento vaginale dopo un ciclo di 5 giorni di terapia orale con progesterone
D. Scansione DEXA che mostra osteopenia

**TI4.**
Una donna di 29 anni G0 P0 con una diagnosi di PCOS è stata interrogata sui pericoli della sua condizione. In particolare, viene messa in guardia sulla possibilità di sviluppare la sindrome metabolica. Quale delle seguenti è la conseguenza più significativa della sindrome metabolica?

A. Ipertiroidismo
B. Malattie cardiovascolari
C. Cancro al seno
D. Insufficienza renale

**TI5.**
Una bambina di 6 anni è nota per avere sviluppo del seno e spotting vaginale. Non si nota alcuna crescita anomala dei capelli. Una massa ovarica di 10 cm viene palpata all'esame rettale. Quale delle seguenti è la diagnosi più probabile?

1.Tumore cistico benigno (dermoide)
2.Idiopatico pubertà precoce
3.Tumore a cellule di Sertoli-Leydig
4.Iperplasia surrenalica congenita
5.Granulosa-thecacelltumor

**TI6.**
Una donna di 35 anni G2 P1001 viene visitata per la sua prima visita prenatale. In base al suo ultimo periodo mestruale, è a 15 settimane di gestazione. Non ha lamentele e non ha una storia medica significativa. Nega la disuria o l'urgenza urinaria. La sua storia chirurgica è notevole solo per turbinectomia da bambina. Il suo ultimo parto è stato un parto vaginale ed è stato senza complicazioni. Ha fatto il Pap test ogni anno che, a sua memoria, "è stato normale". All'esame, è una femmina bianca di bell'aspetto e senza problemi ma con lo smalto sulle unghie di colore rosso . La sua pressione sanguigna (BP) è di 100/65 mm Hg, la frequenza cardiaca (FC) di 90 battiti al minuto (bpm), la frequenza respiratoria (RR) di 12 respiri al minuto, la temperatura di 98°F (36,6°C), il peso di 130 libbre. Il suo esame fisico generale è normale. Il seno non è dolente e non presenta masse o alterazioni cutanee. Il cuore rivela un soffio di eiezione sistolica II/VI. I polmoni sono puliti. Il suo addome non è dolente e la sua altezza del fondo è a livello dell'ombelico. I toni cardiaci fetali sono di 140 bpm. L'esame pelvico rivela genitali esterni normali, vagina e cervice dall'aspetto normale. L'esame bimanuale mostra un'adeguata pelvimetria e utero non dolente senza annessiali o altre masse. La cervice è di consistenza normale e senza masse. Le sue estremità sono prive di edema. I laboratori prenatali sono ottenuti e rivelano quanto segue: CBC: Hgb 10,0 g/dL MCV 82 fL Plt 150.000 WBC 8.000 Rosolia: non immune Gruppo sanguigno: O, Rh negativo HIV ELISA: negativo RPR: negativo
Dosaggio della gonorrea: negativo Antigene di superficie dell'epatite B: positivo
Coombs indiretto (screening anticorpale): negativo UC&S: 10.000 ufc/mL di streptococco di gruppo B Pap test: ASC-US
Test della clamidia: negativo.

Quali elementi devono essere elencati nell'elenco dei problemi? Più scelte:

1. Età materna avanzata (AMA)
   2.età pari o superiore a 35 anni al momento stimato del parto
   3.Dimensione maggiore dell’addome: l'altezza del fondo all'ombelico corrisponde a 20 settimane
   4.Anemia microcitica lieve (Hgb < 10,5)
   5.Antigene di superficie dell'epatite B (HBsAg) positivo Gruppo sanguigno Rh-negativo con Coomb indiretto negativo
   6.Urinocoltura con GBS 10.000 ufc/mL, asintomatica
   7.Pap test che mostra cellule squamose atipiche di significato indeterminato (ASC-US)
   8.Rosolia non immune
   9.Colore dello smalto rosso

**TI7.**
Qual è il tuo prossimo passo per i problemi elencati? Più scelte
1.AMA: consulenza genetica e offerta di amniocentesi genetica
2.Dimensioni/date: ecografia fetale per valutare l'età gestazionale,
3.gestazione multipla
4.Anemia: integrazione con ferro
5.HBsAg positivo: controllare i test di funzionalità epatica e la sierologia dell'epatite B per valutare l'epatite attiva rispetto allo stato di portatore cronico
6.Rh negativo con Coombs negativo indiretto: Rhogam a 28 settimane e al momento del parto se il bambino si rivela Rh positivo
7.Urinocoltura con GBS: trattare con ampicillina e ricoltura delle urine, profilassi con penicillina IV durante il travaglio
8.Pap test ASC-US: osservare e ripetere il Pap test dopo il parto
9.Stato della rosolia: vaccinare dopo il parto
10.Toglierlo lo smalto dalle unghie
11.Consigliarle una consulenza di armocromia

**TI8.**
Una donna di 51 anni lamenta una storia di 4 anni di prurito alla vagina. Si gratta l'area ogni giorno e riferisce che il prurito peggiora di notte. Ha il diabete, ben controllato, è in postmenopausa da 3 anni, nega qualsiasi malattia sessualmente trasmissibile o storia anormale di Pap test e ha quattro figli partoriti per via vaginale. All'ispezione e all'esame dei genitali femminili esterni si rivela quanto segue: genitali femminili esterni dall'aspetto atrofico, il tessuto sopra le piccole labbra è bianco e sottile, il clitoride è difficile da apprezzare, si notano escoriazioni sulle grandi labbra bilaterali e si notano alcuni piccoli lividi all'introito. All'esame è molto tenera ed è difficile inserire uno speculum in quanto l'introito sembra ristretto. La cervice viene visualizzata e non si nota alcuna secrezione. L'esame bimanuale rivela un utero piccolo e non si apprezzano masse annessiali.

Qual è la diagnosi più probabile?
1 Lichen sclerosus
2 Cancro vulvare
3 Candida
4 Herpes

**TI9.**
Rispetto al caso clinico precendente, Qual è il prossimo passo per fare la diagnosi?

1 Biopsia delle aree interessate.
2 La diagnosi è macroscopica
3 Isteroscopia
4 Ecografia pelvica

**TI10.**
Quale delle seguenti affermazioni su T pallidum è corretta?

1.È un protozoo.
2.Se si è infetti, è sconsigliato il concepimento
3.Provoca malattie neonatali principalmente per inoculazione diretta del bambino dalla lesione genitale.
4. I trattamenti alternativi includono la doxiciclina e l'eritromicina.

STROBE Statement—Checklist of items that should be included in reports of ***cross-sectional studies***

|  | Item No | Recommendation |
| --- | --- | --- |
| **Title and abstract** | 1 checked | (*a*) Indicate the study’s design with a commonly used term in the title or the abstract |
|  |  | (*b*) Provide in the abstract an informative and balanced summary of what was done and what was found |
| Introduction | | |
| Background/rationale | 2  checked | Explain the scientific background and rationale for the investigation being reported |
| Objectives | 3  checked | State specific objectives, including any prespecified hypotheses |
| Methods | | |
| Study design | 4  checked | Present key elements of study design early in the paper |
| Setting | 5  checked | Describe the setting, locations, and relevant dates, including periods of recruitment, exposure, follow-up, and data collection |
| Participants | 6  checked | (*a*) Give the eligibility criteria, and the sources and methods of selection of participants |
| Variables | 7  Checked | Clearly define all outcomes, exposures, predictors, potential confounders, and effect modifiers. Give diagnostic criteria, if applicable |
| Data sources/ measurement | 8*  Checked | For each variable of interest, give sources of data and details of methods of assessment (measurement). Describe comparability of assessment methods if there is more than one group |
| Bias | 9  checked | Describe any efforts to address potential sources of bias |
| Study size | 10  Not applicable | Explain how the study size was arrived at |
| Quantitative variables | 11  Checked | Explain how quantitative variables were handled in the analyses. If applicable, describe which groupings were chosen and why |
| Statistical methods | 12  checked | (*a*) Describe all statistical methods, including those used to control for confounding |
|  |  | (*b*) Describe any methods used to examine subgroups and interactions |
|  |  | (*c*) Explain how missing data were addressed |
|  |  | (*d*) If applicable, describe analytical methods taking account of sampling strategy |
|  |  | (*e*) Describe any sensitivity analyses |
| Results | | |
| Participants | 13*  checked | (a) Report numbers of individuals at each stage of study—eg numbers potentially eligible, examined for eligibility, confirmed eligible, included in the study, completing follow-up, and analysed |
|  |  | (b) Give reasons for non-participation at each stage |
|  |  | (c) Consider use of a flow diagram |
| Descriptive data | 14*  checked | (a) Give characteristics of study participants (eg demographic, clinical, social) and information on exposures and potential confounders |
|  |  | (b) Indicate number of participants with missing data for each variable of interest |
| Outcome data | 15*  checked | Report numbers of outcome events or summary measures |
| Main results | 16  checked | (*a*) Give unadjusted estimates and, if applicable, confounder-adjusted estimates and their precision (eg, 95% confidence interval). Make clear which confounders were adjusted for and why they were included |
|  |  | (*b*) Report category boundaries when continuous variables were categorized |
|  |  | (*c*) If relevant, consider translating estimates of relative risk into absolute risk for a meaningful time period |
| Other analyses | 17  checked | Report other analyses done—eg analyses of subgroups and interactions, and sensitivity analyses |
| Discussion | | |
| Key results | 18  Checked | Summarise key results with reference to study objectives |
| Limitations | 19  checked | Discuss limitations of the study, taking into account sources of potential bias or imprecision. Discuss both direction and magnitude of any potential bias |
| Interpretation | 20  Checked | Give a cautious overall interpretation of results considering objectives, limitations, multiplicity of analyses, results from similar studies, and other relevant evidence |
| Generalisability | 21  checked | Discuss the generalisability (external validity) of the study results |
| Other information | | |
| Funding | 22  Not applicable | Give the source of funding and the role of the funders for the present study and, if applicable, for the original study on which the present article is based |

*Give information separately for exposed and unexposed groups.

**Note:** An Explanation and Elaboration article discusses each checklist item and gives methodological background and published examples of transparent reporting. The STROBE checklist is best used in conjunction with this article (freely available on the Web sites of PLoS Medicine at http://www.plosmedicine.org/, Annals of Internal Medicine at http://www.annals.org/, and Epidemiology at http://www.epidem.com/). Information on the STROBE Initiative is available at www.strobe-statement.org.
