# Supplementary material for: The PERFORM Study: Artificial Intelligence Versus Human Residents in Cross-Sectional Obstetrics-Gynecology Scenarios Across Languages and Time Constraints
Source: Mayo Clin Proc Digit Health. 2025 Mar 8;3(2):100206. doi: 10.1016/j.mcpdig.2025.100206 (PMC12190988; doi:10.1016/j.mcpdig.2025.100206)
Supplement: Supplementary Document S2 [file mmc2.pdf]

Supplementary Document S2: “Test Administration and Response Collection Protocol.”

The assessment protocol followed two distinct procedures for human participants and AI systems to ensure standardized evaluation conditions while accounting for their inherent operational differences.

The resident evaluation was conducted in a controlled environment under examination conditions. Prior to testing, participants viewed a standardized instructional video detailing test regulations (available upon request). The assessment was administered in a single room with adequate spacing between participants, where clinical scenarios were projected sequentially. Responses were collected through a digital module under the supervision of two referees. To minimize potential bias, residents were not informed about the question structure or timing parameters beforehand. The informed consent process specified a maximum three-hour duration for clinical scenario responses, with participants retaining the right to withdraw at any time.

AI system assessment was conducted by an obstetrics-gynecology specialist using a MacBook Air M2 with standard internet connectivity in Philadelphia, PA, USA, replicating typical end-user access conditions. The evaluation was completed in two phases: initial testing of Meta AI, Google Gemini, and GPT-3.5 in May 2024, followed by assessments of GPT-4, GPT-4 Mini, Claude 3.5 Sonnet, Claude 3.0 Haiku, and ChatGPT-01 Preview in November 2024.

To ensure response standardization, each AI system received identical prompting instructions. The systems were directed to function as medical practitioners reviewing obstetric and gynecological scenarios, with explicit instructions to provide only the selected answer choice without supplementary commentary. The standardized prompt format was:

“Hi! I'd like you to act as an OB-GYN doctor. I'll show you a clinical case, then you have to answer to a multiple choice questions and I'd just want you to pick the best answer from the choices given. Please just tell me the right answer, do not tell me anything else, do not give any other explanations. Here's the clinical case: [Clinical scenario text]”.
