# Supplementary material for: The PERFORM Study: Artificial Intelligence Versus Human Residents in Cross-Sectional Obstetrics-Gynecology Scenarios Across Languages and Time Constraints
Source: Mayo Clin Proc Digit Health. 2025 Mar 8;3(2):100206. doi: 10.1016/j.mcpdig.2025.100206 (PMC12190988; doi:10.1016/j.mcpdig.2025.100206)
Supplement: Supplementary document S3 [file mmc3.pdf]

This supplementary document (SD3: “Clinical Decision-Making Response Database”) contains a comprehensive collection of binary performance data for both artificial intelligence models and obstetrics-gynecology residents across a set of standard clinical scenarios. Each scenario is indexed with a unique question code. Specifically, FE1 corresponds to Q1; FE2 to Q2; FE3 to Q3; FE4 to Q4; FE5 to Q5; FE6 to Q6; FE7 to Q7; FE8 to Q8; FE9 to Q9; FE10 to Q10; FE11 to Q11; FE12 to Q12; FE13 to Q13; FE14 to Q14; FE15 to Q15; FE16 to Q16; FE17 to Q17; FE18 to Q18; FE19 to Q19; FE20 to Q20; TE1 to Q21; TE2 to Q22; TE3 to Q23; TE4 to Q24; TE5 to Q25; TE6 to Q26; TE7 to Q27; TE8 to Q28; TE9 to Q29; TE10 to Q30; FI1 to Q31; FI2 to Q32; FI3 to Q33; FI4 to Q34; FI5 to Q35; FI6 to Q36; FI7 to Q37; FI8 to Q38; FI9 to Q39; FI10 to Q40; FI11 to Q41; FI12 to Q42; FI13 to Q43; FI14 to Q44; FI15 to Q45; FI16 to Q46; FI17 to Q47; FI18 to Q48; FI19 to Q49; FI20 to Q50; TI1 to Q51; TI2 to Q52; TI3 to Q53; TI4 to Q54; TI5 to Q55; TI6 to Q56; TI7 to Q57; TI8 to Q58; TI9 to Q59; TI10 to Q60.

Each respondent’s performance is represented as a sequence of 60 binary digits, with “1” indicating a correct response and “0” indicating an incorrect response. By referencing the question code mapping, each binary digit can be attributed to a specific scenario. These data enable quantitative analyses of diagnostic accuracy, comparative performance assessments among different models and training levels, and evaluation of reasoning patterns over time.

The following are the provided sequences of responses, each line corresponding to a single respondent’s performance on the 60 scenarios.

AI models

AI-meta: 1 1 1 1 1 0 1 1 1 0 1 1 1 0 1 1 0 1 0 1 1 1 1 1 1 0 0 1 0 1 1 1 1 1 1 1 1 0 0 1 0 0 1 1 0 1 1 0 0 1 1 1 0 1 1 0 1 1 1 0

AI-gemini: 1 1 1 1 0 0 1 1 1 0 1 1 1 1 1 1 1 1 1 1 1 1 1 1 1 0 0 1 0 1 0 1 1 1 1 0 1 0 1 1 0 0 1 1 1 1 1 0 1 1 0 0 0 1 0 0 1 1 1 1

AI-chat gpt 3.5: 0 1 0 1 0 1 1 1 1 0 1 1 1 1 1 1 1 1 1 1 1 1 1 1 1 1 0 0 0 1 1 1 1 1 1 0 0 0 1 1 0 0 1 1 1 1 1 0 0 0 0 0 1 1 0 0 1 1 0

AI-chat gpt4o: 1 1 1 1 1 1 1 1 1 0 1 1 1 1 1 1 1 1 1 1 1 1 1 1 1 1 0 0 1 1 1 1 1 1 1 1 1 1 0 0 1 1 1 1 1 0 1 1 0 0 1 1 1 1 1 1 1 1 1 1

AI-chat gpt 4mini: 1 1 1 1 1 0 1 1 1 0 1 1 1 0 1 1 1 1 1 1 1 1 1 1 1 0 0 0 1 0 1 1 1 1 0 1 0 0 0 1 0 1 1 0 0 0 0 0 0 0 0 0 1 1 0 0 0 1 1 0

AI-claude sonnet 3.5: 1 1 1 1 0 1 1 1 1 0 1 1 1 1 1 1 1 1 1 1 1 1 1 1 1 0 0 1 0 1 1 1 1 1 1 1 1 1 0 0 1 1 1 1 1 1 1 1 0 0 1 1 1 1 1 1 1 0 1 1 1 1

AI-claude 3.0 haiku: 1 1 0 1 1 1 1 1 1 0 1 1 1 0 1 1 1 1 1 1 1 0 1 1 1 1 0 1 0 1 1 1 1 0 1 1 1 1 0 1 0 1 1 1 0 1 1 0 0 0 0 0 0 0 0 0 0 1 0 1

AI-chat gpt 01-preview: 1 1 1 1 1 1 1 1 1 0 1 1 1 1 1 1 1 1 1 1 1 1 1 1 1 1 0 1 1 1 1 1 1 1 1 1 1 1 0 1 1 1 1 1 1 0 1 1 1 0 1 0 1 1 1 1 1 1 1 1 1

Obstetrics-gynecology residents:

First year resident: 1 1 1 1 0 0 1 1 1 0 1 1 1 0 1 1 1 1 1 0 0 1 1 1 1 0 1 1 0 0 0 1 1 1 0 1 1 0 0 1 1 0 1 1 0 0 1 0 1 0 0 0 0 1 0 0 0 1 1 0

First year resident: 1 1 1 1 0 1 1 1 1 0 1 1 1 0 1 1 1 1 1 0 0 1 1 1 1 0 0 1 0 0 0 1 1 1 0 1 1 0 0 1 1 0 1 1 0 1 1 0 1 0 0 0 0 1 0 0 0 1 1 0

First year resident: 1 1 1 1 0 0 1 1 1 0 1 1 1 1 1 1 1 1 1 0 0 1 1 1 1 0 0 1 0 0 0 1 1 1 0 1 1 0 0 1 1 0 1 1 0 0 1 0 1 0 0 0 0 1 0 0 0 1 1 0

First year resident: 1 0 1 0 1 0 1 0 1 0 1 0 1 0 1 1 1 0 1 0 1 1 1 0 1 0 0 1 1 0 1 1 1 0 1 1 1 0 0 1 1 0 1 0 0 1 0 0 0 0 0 0 0 0 1 1 0 0 0 0 0

First year resident: 1 1 1 1 1 0 1 0 1 0 1 0 1 0 1 0 1 0 1 0 0 1 1 0 1 0 0 1 0 0 0 1 1 1 1 1 1 0 0 0 1 0 1 1 0 1 0 0 1 0 0 0 0 1 1 0 0 1 1 0

Second year resident: 1 1 0 0 0 0 1 1 1 0 1 1 1 1 1 1 0 1 1 0 0 1 0 0 0 0 0 0 0 0 1 1 1 0 0 1 0 0 0 1 1 0 1 1 0 1 0 0 1 0 0 0 0 1 1 0 0 1 1 0

Second year resident: 1 1 1 0 0 1 1 1 1 1 1 1 1 1 1 1 1 1 1 0 0 1 1 1 1 0 1 1 0 0 0 1 1 1 1 1 1 0 0 1 1 0 1 1 0 1 1 0 0 0 0 0 0 0 1 0 0 0 1 1 0

Second year resident: 0 1 1 1 0 1 1 1 1 0 1 1 1 0 1 1 1 1 1 0 0 1 1 0 1 0 0 0 1 0 1 1 1 1 0 1 1 0 1 1 1 0 1 0 0 1 0 0 1 0 0 0 0 1 1 0 0 1 0 0

Second year resident: 1 1 1 1 1 1 1 1 1 0 1 1 1 0 1 1 1 0 1 0 0 0 1 0 1 0 0 0 1 0 1 1 1 1 1 1 1 1 0 1 1 1 0 1 1 0 1 0 0 1 0 0 0 0 1 1 0 0 1 1 0

Second year resident: 0 0 1 0 1 0 0 1 1 0 1 0 1 1 1 1 1 1 1 0 0 1 1 0 1 0 0 0 0 0 1 1 1 0 1 0 1 1 0 1 1 0 1 1 0 1 0 0 0 0 0 0 0 0 1 0 0 0 1 1 0

Second year resident: 0 0 0 1 1 0 1 1 1 0 1 1 1 1 1 1 1 1 1 0 0 1 1 0 0 0 1 0 0 0 0 1 0 1 1 1 1 1 0 1 1 0 1 1 1 1 0 0 1 1 1 0 0 1 1 0 0 1 1 1

Second year resident: 0 1 1 1 0 1 1 1 1 0 1 1 1 0 1 1 1 1 1 0 0 0 1 0 1 0 0 0 1 0 1 1 1 1 1 1 1 0 1 1 1 0 1 1 0 1 0 0 1 0 0 0 0 1 1 0 0 1 1 0

Third year resident: 1 1 0 1 0 0 0 1 1 0 1 1 1 1 0 1 1 1 1 1 0 0 1 1 1 0 1 1 0 1 1 1 1 1 1 1 1 1 0 1 1 0 0 1 1 1 1 0 0 1 1 1 0 0 1 1 0 1 1 1 1

Third year resident: 1 1 1 1 0 1 1 1 1 0 1 1 1 0 1 1 1 1 1 0 0 1 1 1 1 0 0 1 0 1 0 1 1 1 1 0 0 0 0 1 1 0 1 1 0 1 1 0 0 0 0 0 0 1 0 0 0 1 1 0

Third year resident: 0 1 1 0 0 0 1 1 1 0 1 1 1 0 0 1 1 1 1 0 0 1 1 0 1 0 1 1 0 1 0 1 1 0 1 1 0 0 1 1 0 0 1 1 1 1 0 0 1 0 1 0 1 1 1 0 0 1 1 0

Third year resident: 1 0 1 1 1 1 1 0 1 0 0 1 1 1 1 1 1 1 1 1 0 0 1 0 1 0 0 0 0 1 1 1 0 1 0 1 1 0 1 1 1 0 1 0 1 1 1 0 1 0 1 0 0 1 1 0 0 1 1 0

Fourth year resident: 1 1 1 1 0 1 1 1 1 0 1 1 1 0 1 1 1 1 1 0 0 0 1 0 1 0 0 0 1 0 1 1 1 1 1 1 1 0 1 1 1 0 1 1 0 0 0 0 1 0 0 0 0 1 1 0 0 1 1 0

Fourth year resident: 1 1 1 1 0 1 1 1 1 0 1 1 1 1 1 1 1 1 1 1 0 1 1 0 0 0 0 1 0 1 1 1 1 0 1 1 1 0 0 1 1 0 1 1 0 1 0 0 1 1 1 0 0 1 1 0 0 1 1 1

Fourth year resident: 1 1 1 1 1 1 1 1 1 0 1 1 1 0 1 1 1 1 1 1 0 1 1 1 1 1 0 1 0 1 1 1 1 1 1 1 1 1 0 1 1 1 0 1 1 0 1 1 1 1 1 0 1 0 1 1 0 1 1 1 1

Fourth year resident: 1 1 1 1 1 1 1 1 1 0 1 1 1 0 1 1 1 1 1 1 0 1 1 1 1 1 0 1 0 1 1 1 1 1 1 1 1 1 1 1 1 1 0 1 1 1 1 1 1 1 1 1 0 1 1 1 1 0 1 1 1

Fifth year resident: 1 1 1 1 1 1 1 1 1 0 1 1 1 0 1 1 1 1 1 1 0 1 1 1 1 1 0 1 0 1 1 1 1 1 1 1 1 1 0 1 1 1 0 1 1 0 1 1 1 1 1 1 0 0 0 1 1 0 0 1 1 1

Fifth year resident: 1 1 1 1 1 1 1 1 1 1 1 1 1 1 1 1 1 1 1 1 1 1 1 1 1 1 1 1 0 1 0 1 1 1 1 1 1 1 1 1 0 1 1 1 0 1 1 0 1 1 1 1 1 1 0 0 0 1 1 0 0 1 1 1

Fifth year resident: 1 1 1 1 1 1 1 1 1 1 1 1 1 1 1 1 1 1 1 1 1 1 1 1 1 1 1 1 0 1 1 1 1 1 1 1 1 1 1 1 1 1 1 1 1 0 1 1 0 1 1 1 1 1 1 0 1 0 1 1 1 0 1 1 1

Fifth year resident: 1 1 1 1 1 1 1 1 1 1 1 1 1 1 1 1 1 1 1 1 1 1 1 1 1 1 1 1 1 1 0 1 1 1 1 1 1 1 1 1 1 1 1 1 1 1 1 1 1 1 1 1 1 1 1 1 0 1 1 1 1 1 1 1

These data, when read in conjunction with the assigned question codes, facilitate a granular understanding of diagnostic accuracy for each respondent. Each “1” or “0” can be traced back to

a specific clinical scenario, and aggregated data can be analyzed to identify patterns, strengths, and areas in need of improvement. This format supports statistical methods for comparing cognitive reasoning across different AI models and various levels of clinical training.
