# Supplementary material for: The PERFORM Study: Artificial Intelligence Versus Human Residents in Cross-Sectional Obstetrics-Gynecology Scenarios Across Languages and Time Constraints
Source: Mayo Clin Proc Digit Health. 2025 Mar 8;3(2):100206. doi: 10.1016/j.mcpdig.2025.100206 (PMC12190988; doi:10.1016/j.mcpdig.2025.100206)
Supplement: Supplementary document S4 [file mmc4.pdf]

Supplementary Document S4 "Technical Framework and Statistical Analysis Protocol for AI Integration in Obstetric Decision-Making".

A. Technical Analytical Framework and Statistical Methodology

Our analytical framework employed systematic statistical methodologies to evaluate artificial intelligence implementation in obstetric clinical decision-making. Each endpoint utilized specific analytical protocols to ensure robust evaluation of system performance and integration feasibility.

Primary Outcome Analysis Performance comparison between AI systems and residents utilized two-proportion Z-tests with Wilson confidence intervals for precision estimation. Effect size quantification employed odds ratio calculations and Cohen's d metrics, with statistical significance threshold set at $\alpha=0.05$.

Secondary Endpoint Analysis

1. AI System Performance Stratification: Inter-platform performance variations were assessed through chi-square analysis (χ^2) with post-hoc Fisher's Exact Tests incorporating false discovery rate correction. Performance tier classification utilized standardized thresholds (high ≥80%, medium 70-80%, low <70%), with fold-change calculations quantifying relative performance differentials.
2. Resident Expertise Development: Longitudinal performance assessment employed one-way ANOVA for between-year comparisons. Expertise development quantification utilized Gini coefficients and entropy calculations, supplemented by compound annual growth rate (CAGR) analysis for temporal progression assessment.
3. Linguistic Impact: The analytical protocol comprised three distinct phases:
    a. Performance metrics calculation across linguistic domains (30 English, 30 Italian scenarios)
    b. Differential impact quantification through independent t-tests ($\alpha=0.05$)
    c. Stratified analysis of high-performing (top third) versus mid-to-low performing participants
4. Temporal Pressure Effects: Assessment utilized paired t-tests and Mann-Whitney U analysis for between-group comparisons. Difficulty-adjusted analysis employed Wilcoxon signed-rank testing (n=24) with standardized effect size calculations using AI performance as baseline metric.
5. Complexity Assessment: Question complexity evaluation implemented the Flesch Reading Ease Formula (range: 0-100) across balanced scenario distribution.

Performance evaluation utilized binary scoring methodology (1=correct, 0=incorrect) for standardized assessment.

6. Error Pattern Analysis: Analysis focused on high-performing AI systems (≥80% accuracy) through:
    a. Performance baseline assessment using mean accuracy inverse calculations
    b. Pearson correlation coefficient analysis with regression parameters
    c. Stratified analysis across training levels using one-sample t-tests
    d. Effect size quantification through Cohen's d calculations
7. Integration Potential Assessment: Implementation feasibility analysis incorporated:
    a. Baseline performance characterization of high-performing AI systems
    b. Integration impact assessment through one-sample t-tests
    c. Degradation rate quantification across 300 integration scenarios
    d. Between-year ANOVA with pairwise comparisons
8. Response Consistency: Cognitive stability assessment utilized paired clinical scenarios in both languages under varying temporal conditions. Analysis employed Fisher's Exact Tests for between-group comparisons with binary response matching for concordance calculations.

B. Computational Environment and Software

The computational analysis framework was implemented using Python programming language (version 3.11.6, packaged by conda-forge) within Jupyter Notebook environments, establishing a standardized and reproducible analytical pipeline. Our analysis infrastructure incorporated essential scientific computing libraries, selected for their specific analytical capabilities and documented performance characteristics.

The core analytical framework utilized pandas (version 2.2.3) for data manipulation and cleaning operations, while numpy (version 1.24.4) provided the foundation for numerical computations. Statistical analyses were conducted through scipy (version 1.11.3), with emphasis on parametric and non-parametric testing methodologies. Data visualization and analytical representation employed matplotlib (version 3.8.0) and seaborn (version 0.13.0). Machine learning operations, including hierarchical clustering analyses, were executed through scikit-learn (version 1.3.1).

The computational environment was standardized using conda-forge package management to ensure analytical consistency. This standardization protocol supported reliable replication of computational procedures across different research environments while maintaining version-specific functionality.

Our data processing pipeline followed a systematic approach to ensure data integrity. Initial data preparation involved cleaning procedures using pandas frameworks to address missing values, standardize data formats, and correct entry errors across both AI-generated and resident-provided responses. Performance metric standardization was achieved through numpy-based normalization techniques, enabling direct comparison between AI systems and human participants while accounting for variations in question complexity.

Agreement analysis between AI and human responses used established statistical methodologies, including Cohen's kappa coefficients and percentage agreement calculations, implemented through scipy's statistical functions. These metrics provided quantitative assessment of response concordance while accounting for chance agreement.

The complete codebase and methodological documentation are available upon request to support independent verification and replication of all analytical procedures and findings.
