# Supplementary material for: The PERFORM Study: Artificial Intelligence Versus Human Residents in Cross-Sectional Obstetrics-Gynecology Scenarios Across Languages and Time Constraints
Source: Mayo Clin Proc Digit Health. 2025 Mar 8;3(2):100206. doi: 10.1016/j.mcpdig.2025.100206 (PMC12190988; doi:10.1016/j.mcpdig.2025.100206)
Supplement: Supplementary Document S5 [file mmc5.pdf]

Supplementary Document S5: "complexity score assessment for GYN-OB questions"

The complexity assessment methodology for gynecological-obstetric clinical scenarios employs a multi-dimensional approach centered on the Flesch Reading Ease Score, while incorporating additional contextual factors specific to clinical decision-making environments. The scoring framework integrates both quantitative linguistic metrics and qualitative clinical parameters to generate comprehensive complexity indices. The primary complexity metric utilizes the Flesch Reading Ease formula: Score = 206.835 - (1.015 × ASL) - (84.6 × ASW), where ASL represents Average Sentence Length and ASW denotes Average Syllables per Word. This baseline calculation provides an initial complexity assessment ranging from 0 to 100, with lower scores indicating higher complexity levels. The interpretation framework segments scores into clinically relevant categories: very difficult (0-30, appropriate for specialized medical knowledge), difficult (30-50, suitable for advanced clinical training), fairly difficult (50-60, appropriate for senior medical students), and standard to easy (60-100, accessible to general medical practitioners). Our analysis revealed distinct complexity patterns across linguistic domains and temporal constraints. English-language scenarios demonstrated higher mean complexity scores (61.61) compared to Italian scenarios (38.88), suggesting significant variations in cognitive load across linguistic contexts. This differential may reflect both inherent linguistic structures and the specialized medical terminology requirements in each language. Temporal constraints showed minimal impact on complexity metrics (free-timing: 49.16; time-constrained: 52.42), indicating that scenario complexity remains relatively stable regardless of time pressure. The complexity distribution analysis revealed notable positive skewness (3.59), suggesting a preponderance of moderately complex scenarios with selective inclusion of highly complex clinical presentations. This distribution aligns with typical clinical practice patterns, where routine cases constitute the majority of encounters while maintaining representation of more challenging scenarios that test advanced clinical reasoning capabilities. This comprehensive scoring approach provides a validated framework for evaluating scenario complexity across both artificial intelligence systems and human practitioners, enabling more nuanced understanding of performance patterns in clinical decision-making contexts.
