# Supplementary material for: The PERFORM Study: Artificial Intelligence Versus Human Residents in Cross-Sectional Obstetrics-Gynecology Scenarios Across Languages and Time Constraints
Source: Mayo Clin Proc Digit Health. 2025 Mar 8;3(2):100206. doi: 10.1016/j.mcpdig.2025.100206 (PMC12190988; doi:10.1016/j.mcpdig.2025.100206)
Supplement: Supplementary Document SD6 [file mmc6.docx]

**Supplementary Document : “SD6 - Informed Consent”**

Informed Consent Form

Introduction

This document serves as the informed consent form for participation in a scientific study designed to assess medical diagnostic skills through multiple-choice questions. Participation in this trial is entirely voluntary, and all responses will be anonymized to ensure confidentiality. This form provides all the necessary information about the trial so you can make an informed decision regarding your participation.

Purpose of the Trial

The primary objective of this trial is to evaluate the diagnostic abilities of medical residents using a structured format of multiple-choice questions. The study aims to gather data on decision-making processes and test knowledge in a controlled environment, contributing to research on medical education and diagnostic practices.

Procedures

You will answer a series of multiple-choice questions related to various medical scenarios.

You are required to use your personal laptop to access and answer the questions. Please ensure your device is in good working condition and that you have a reliable internet connection for the duration of the session.

The session will last approximately 2 hours, conducted in a supervised environment.

You are allowed to take short breaks if needed but are expected to complete the session within the allotted time unless you choose to withdraw.

You may discontinue your participation at any point without any consequences.

Potential Risks and Discomforts

There are no risks associated with participating in this trial since you can discontinue your participation at any point for any reason. No physical risks are involved.

Benefits

While there are no direct benefits to you, your participation will contribute valuable data to the scientific community, potentially improving medical training and diagnostic procedures. You may also find the reflective nature of the questions to be an informative review of your knowledge and decision-making skills.

Confidentiality

All information collected during this trial will be strictly confidential. Your answers will be anonymized, and no personal identifying information will be linked to your responses. Data will be stored in a secure database accessible only to the research team for analysis purposes. Study results may be published, but participants will not be identifiable in any reports or publications.

Voluntary Participation and Withdrawal

Your participation in this trial is completely voluntary. You have the right to withdraw from the trial at any time without penalty. If you decide to withdraw, any data collected will be excluded from the study upon your request.

Declaration of Language Proficiency

By signing this consent form, you declare that you are proficient in both Italian and English, which will be used to present the questions and instructions during the trial.

Declaration of Equipment

By signing this consent form, you confirm that you will use your personal laptop for the duration of the trial. You acknowledge that you are responsible for ensuring that your equipment is suitable for use and that you have adequate technical support if any issues arise.

Acknowledgment

Thank you for considering participation in this trial. Your contribution is invaluable to advancing our understanding of medical diagnostic skills and will potentially impact the improvement of educational strategies in the medical field. We appreciate your time and effort in helping us conduct this important research.

Consent Statement

I have read this informed consent form and have had the opportunity to ask questions about the study. All my questions have been answered to my satisfaction, and I understand the conditions of my participation. By signing below, I agree to participate in the trial under the terms outlined above.

All participants signed the consent form before taking part, confirming their understanding of the study’s purpose, procedures, data handling, and voluntary nature. No personal identifiers were linked to their responses; hence, all data collected were completely anonymized. Signed informed consent forms are securely stored and may be made available to reviewers upon reasonable request, in compliance with data protection regulations. The study will be carried out in line with the institutional guidelines of the University of Messina and the applicable Italian regulations for minimal-risk, observational research.
